# Analytical Methods for Allergen Control in Food Processing

**DOI:** 10.3390/foods12071439

**Published:** 2023-03-28

**Authors:** Nanju Alice Lee, Andreas Ludwig Lopata, Michelle Lisa Colgrave

**Affiliations:** 1 School of Chemical Engineering, University of New South Wales, Sydney, NSW 2052, Australia; 2 Centre for Food and Allergy Research, Murdoch Children’s Research Institute, Parkville, VIC 3052, Australia; 3 Australian Institute of Tropical Health and Medicine, James Cook University, Douglas, QLD 4811, Australia; 4 Molecular Allergy Research Laboratory, College of Public Health, Medical and Veterinary Sciences, James Cook University, Douglas, QLD 4811, Australia; 5 ARC Centre of Excellence for Innovations in Peptide and Protein Science, School of Science, Edith Cowan University, Joondalup, WA 6027, Australia

Food allergy and food-related anaphylaxis have become a growing public health and food safety issue worldwide. The World Allergy Organization (WAO) estimated that 220–250 million people would live with food allergies, based on the reported prevalence of 5–8% in children and 1–2% in adults [1]. Without a practical treatment and cure for food allergy currently available, the diligent avoidance of allergenic foods together with the prompt treatment of symptoms is the best management option available to allergic individuals [2]. As a result, regulatory bodies in many countries mandate food allergen labelling to help allergic consumers to make informed food choices and avoid accidental exposure [3].

Since the publication of the CODEX Alimenterius International Food Standards (CODEX hereafter) recommendation on food allergen labelling in 1999 (CODEX STAN 1-1985) [4], many countries have adopted its recommendation and mandated the labelling of the CODEX-recommended priority allergens or country-specific allergens [3]. From the perspective of industry practice, the allergen labelling framework has come a long way since its establishment in the early 2000s and has undergone significant development, especially in the last 5 years. A particularly noteworthy development was the establishment of industry guidance on allergen management or control, allergen detection methodologies, and precautionary allergen labelling (PAL) in view of helping manage unintentional cross-contacts and establishing method detection-based regulatory levels (e.g., 10 ppm in Japan [5]) or clinical threshold-based reference doses (e.g., Voluntary Incidental Trace Allergen Labelling (VITAL^®^) [6,7]. Although the industry is developing and refining its allergen management practices, practising total avoidance via allergen labels poses challenges for allergic individuals and PAL statements have frequently proven to be inaccurate in both domestic and imported products [8,9,10]. This creates mistrust from both consumers and health-care providers, and they often ignore PAL warnings on food packages [3,11,12,13].

Recognising an urgent need to harmonise food allergen labelling and allergen management or control, CODEX adopted the Code of Practice on Food Allergen Management (CODEX CXC 80-2020) in 2020 (https://www.fao.org/fao-who-codexalimentarius/sh-proxy/en/?lnk=1&url=https%253A%252F%252Fworkspace.fao.org%252Fsites%252Fcodex%252FStandards%252FCXC%2B80-2020%252FCXC_080e.pdf (accessed on 19 March 2023)). In addition, the Food and Agriculture Organization of the United Nations (FAO) and World Health Organization (WHO) jointly convened an expert panel consultation on Risk Assessment of Food Allergens in 2020–2022. The overall aims of this expert panel consultation were to “(i) review and update the list of priority allergens for the labelling of packaged foods based on risk assessment, (ii) establish references doses (based on health-based guidance values) and their corresponding action levels in foods for the priority allergens, and (iii) evaluate the evidence in support of PAL and establish guidance for PAL”. The ad hoc Joint FAO/WHO Expert Consultations on Risk Assessment of Food Allergens was held over four separate meetings and the expert committee decided to only discuss immune-mediated hypersensitivities, such as IgE-mediated food allergies and coeliac disease. The first meeting established the revised list of global priority allergens and a list of national or regional-specific priority allergens, based on global prevalence, severity, and potency assessments (Table 1). Soy has been removed from the priority allergen list but was added to the national or regional-specific allergen list. Sesame has been added as a new priority allergen to the global list. The second meeting established a set of reference doses at ED_05_ as a level of exposure without appreciable risks to health for the recommended priority allergens (Table 1) and the action levels based on food consumption in different types of foods. The second meeting also reviewed the current analytical capability and any limitations in supporting the reference dose-based risk assessment. The third meeting established guidance on the use of PAL to address the unintended allergen presence (UAP) due to cross-contact. The fourth meeting addressed the exemptions. The executive summaries of all four meetings and full reports of the first two meetings can be found at (https://www.fao.org/food-safety/scientific-advice/food-allergens/en/ (accessed on 23 February 2023)). 

The analytical methodology for the detection and quantification of allergen residues in foods is an integral part of the evidenced-based risk assessment for label decisions including precautionary allergen labelling (PAL). Accurate and precise allergen detection, however, continues to be a challenging task due to a high uncertainty of the analysis arising from the natural susceptibility of proteinaceous allergens to food preservation and processing conditions. As concluded by the FAO–WHO expert panel, the current analytical methods could accommodate the implementation of the recommended reference doses and action levels, but limitations still exist with certain analytical methods and allergens in certain forms in processed foods (e.g., protein hydrolysates). 

Against this background, this Special Issue set the theme of “Analytical Methods for Allergen Control in Food Processing”. It contains a collection of 12 papers (10 research papers and 2 review papers) on a wide range of allergens investigating various aspects related to allergen detection. The two review papers [14,15] collectively addressed the need for fit-for-purpose analytical test methods. For example, Tuppo et al. (2022 [15]) reviewed the potential of multiplex microarray immunoassay in the IgE-based assessment of molecular changes on the ability of food products to induce an allergic reaction. Jiang and Rao (2021 [14]) also pointed out an important terminology issue that immunogenicity, antigenicity, and allergenicity of food allergens are not well-defined or understood; thus, the methodologies used to measure each of these terms are sometimes inappropriate from an immunological and clinical viewpoint. This paper reviewed the effects of food processing on fish allergens, a difficult-to-quantify allergen source. 

This Special Issue covers many different allergen detection methodologies; some are more conventional but are still valuable tools and others are newly emerging techniques. Enzyme-linked immunosorbent assay (ELISA) still serves as a gold standard for allergen detection due to its commercial convenience. The ELISA is ideal for detecting intact protein or large fragments (greater than 15 amino acids) but does often not achieve the equivalent detection of structurally changed allergens. Nevertheless, ELISA has been utilized to detect molecular changes in the study to optimize processing conditions for hydrostatic high pressure combined with heat for whey allergens [16] and in the enzymatic hydrolysis and fermentation of pea protein isolate with reduced immunoreactivity [17]. The binding specificity of biorecognition molecules dictates the accuracy and precision in allergen detection and the development of new biorecognition molecules is also emerging. A recombinant single-domain antibody specific to gluten was produced and incorporated into an ELISA, showing a promising result [18]. 

It is envisioned that the area of user-friendly rapid on-site testing allergen sensors will see significant development in future. To this effect, an electrochemical immunosensor for the simultaneous detection of two peanut allergens in food matrices has been demonstrated [19]. Dual sensors helped to increase the sensor detectability of allergens in processed foods. 

PCR methods and particularly mass spectrometry (MS) have gained momentum in recent years. By measuring DNA rather than protein, PCR is an excellent method for difficult-to-measure allergens using an immunochemical approach. However, not all allergens are ideal for the application of PCR methods. A comparative study between ELISA and real-time PCR for the detection of walnut residues in commercial food products [18] concluded that although PCR was more sensitive than ELISA, the two methodologies were comparable in most products. ELISA failed to perform well for thermally processed samples and those products containing pecan, which is closely related to walnut. 

MS is gaining more confidence in allergen detection and quantification. Either by itself or coupled with other methods such as immunoaffinity, for example, as seen in [20], would enhance the performance of MS by improving method sensitivity. Similarly, combining a competitive ELISA and MS allowed for the quantification of gluten in dried yeast and yeast-containing products, as demonstrated in [21]. MS is particularly suited for the detection of peptides or polypeptides of “hydrolysed” allergens. This is well demonstrated in [1], in which IgE epitopes and coeliac toxic motifs are detected in the digesta of soy-enriched wheat-based pizza bases. A new MS method was developed and applied to the analysis of 14 different α-amylase/trypsin inhibitors in wheat [22]. 

In all these methodologies described above, sample preparation remains the most labour-intensive step and innovation is needed to simplify this action with greater efficiency. Appropriate sample preparation, including extraction steps, is critical in allergenic protein discovery. In this regard, Nugraha et al., 2021 [23], reported an extraction method with an optimized extraction buffer to improve the solubility of allergenic proteins from oyster tissue. 

In summary, this Special Issue was successful in attracting the latest research in different innovations directed at improving analytical capacity for allergen detection and quantification. No doubt allergen test methods will continue to play a key role in the successful implementation of FAO–WHO recommended reference doses for priority allergens. We, the editors, are looking forward to new development and innovation in the near future.

## Figures and Tables

**Table 1:** Summary of the FAO–WHO expert panel recommended priority allergen and their reference doses for risk management through food labels.

Global Priority Allergens	Recommended Reference Doses (mg Total Protein from the Allergen Source)
Tree nuts (walnut, pecan, cashew, pistachio and almond)	1.0
Milk	2.0
Peanut	2.0
Egg	2.0
Sesame	2.0
Hazelnut	3.0
Wheat	5.0
Fish	5.0
Shrimp	200
**Nationally or regionally specific allergens**	
Tree nuts (Brazil nut, macadamia, and pine nut)	NR
Buckwheat	NR
Lupin	NR
Mustard	NR
Oats	NR
Soybean	NR

NR = not recommended at the time of this publication.

## Data Availability

The data presented in this editorial are openly available in (1) FAO and WHO. 2022. *Risk Assessment of Food Allergens. Part 1—Review and validation of Codex Alimentarius priority allergen list through risk assessment.* Meeting Report. Food Safety and Quality Series No. 14. Rome at https://doi.org/10.4060/cb9070en, page xv, and (2) FAO. 2022. *Risk Assessment of Food Allergens—Part 2: Review and establish threshold levels in foods for the priority allergens*. Meeting Report. Food Safety and Quality Series No. 15. Rome at https://doi.org/10.4060/cc2946en, page xvii.

## References

[1] Daly M.E., Wang K., Pan X., Depau R.L., Marsh J., Capozzi F., Johnson P., Gethings L.A., Mills E.N.C. (2022). The Fate of IgE Epitopes and Coeliac Toxic Motifs during Simulated Gastrointestinal Digestion of Pizza Base. Foods.

[2] Abrams E.M., Simons E., Gerdts J., Nazarko O., Povolo B., Protudjer J.L.P. (2020). “I Want to Really Crack This Nut”: An Analysis of Parent-Perceived Policy Needs Surrounding Food Allergy. BMC Public Health.

[3] Roche I., Vale S.L., Hornung C.J., Zurzolo G.A., Netting M.J., Dharmage S.C., Gray C., Lee N.A., Lacis-Lee J., Jorgensen P.F. (2022). An International First: Stakeholder Consensus Statement for Food Allergen Management in Packaged Foods and Food Service for Australia and New Zealand. J. Allergy Clin. Immunol. Pract..

[4] (2018). Codex Alimentarius International Food Standards General Standard for the Labelling of Prepackaged Foods. Codex Aliment..

[5] Akiyama H., Adachi R. (2021). Japanese Food Allergy-Labeling System and Comparison with the International Experience; Detection and Thresholds. Food Saf..

[6] Brooke Taylor S., Christensen G., Grinter K., Sherlock R., Warren L. (2018). The Allergen Bureau VITAL Program. J. AOAC Int..

[7] Koeberl M., Clarke D., Allen K.J., Fleming F., Katzer L., Alice Lee N., Lopata A.L., Said M., Scheelings P., Shepherd N. (2018). Food Allergen Management in Australia. J. AOAC Int..

[8] Blom W.M., Michelsen-Huisman A.D., van Os-Medendorp H., van Duijn G., de Zeeuw-Brouwer M.-L., Versluis A., Castenmiller J.J.M., Noteborn H.P.J.M., Kruizinga A.G., Knulst A.C. (2018). Accidental Food Allergy Reactions: Products and Undeclared Ingredients. J. Allergy Clin. Immunol..

[9] Shwe Yee N., Shao Q., Uraipong C., Shoji M., Lee N.A. (2021). A Comprehensive Survey of Allergen Labeling on Pre-Packaged Food Products Imported from Mainland China. Food Control..

[10] Uraipong C., Kaewdang P., Shwe Yee N., Shoji M., Lee N.A. (2021). A Survey of Food Allergen Labeling and Undeclared Allergen Residues in Pre-Packaged Food Products Imported from Thailand. Food Control..

[11] Allen K.J., Turner P.J., Pawankar R., Taylor S., Sicherer S., Lack G., Rosario N., Ebisawa M., Wong G., Mills E.N.C. (2014). Precautionary Labelling of Foods for Allergen Content: Are We Ready for a Global Framework?. World Allergy Organ. J..

[12] Turner P.J., Allen K.J., Mehr S., Campbell D.E. (2016). Knowledge, Practice, and Views on Precautionary Allergen Labeling for the Management of Patients with IgE-Mediated Food Allergy-a Survey of Australasian and UK Health Care Professionals. J. Allergy Clin. Immunol. Pract..

[13] Madsen C.B., van den Dungen M.W., Cochrane S., Houben G.F., Knibb R.C., Knulst A.C., Ronsmans S., Yarham R.A.R., Schnadt S., Turner P.J. (2020). Can We Define a Level of Protection for Allergic Consumers That Everyone Can Accept?. Regul. Toxicol. Pharmacol..

[14] Jiang X., Rao Q. (2021). Effect of Processing on Fish Protein Antigenicity and Allergenicity. Foods.

[15] Tuppo L., Giangrieco I., Tamburrini M., Alessandri C., Mari A., Ciardiello M.A. (2022). Detection of Allergenic Proteins in Foodstuffs: Advantages of the Innovative Multiplex Allergen Microarray-Based Immunoassay Compared to Conventional Methods. Foods.

[16] Sun X., Chua J.V., Le Q.A., Trujillo F.J., Oh M.-H., Campbell D.E., Mehr S., Lee N.A. (2021). A Response Surface Methodology (RSM) Approach for Optimizing the Attenuation of Human Ige-Reactivity to β-Lactoglobulin (β-Lg) by Hydrostatic High Pressure Processing. Foods.

[17] Arteaga V.G., Demand V., Kern K., Strube A., Szardenings M., Muranyi I., Eisner P., Schweiggert-Weisz U. (2022). Enzymatic Hydrolysis and Fermentation of Pea Protein Isolate and Its Effects on Antigenic Proteins, Functional Properties, and Sensory Profile. Foods.

[18] García-García A., Madrid R., Garcia-Calvo E., Mendoza-Chamizo B., García T., Martin R. (2020). Production of a Recombinant Single-Domain Antibody for Gluten Detection in Foods Using the Pichia Pastoris Expression System. Foods.

[19] Freitas M., Neves M.M.P.S., Nouws H.P.A., Delerue-Matos C. (2021). Electrochemical Immunosensor for the Simultaneous Determination of Two Main Peanut Allergenic Proteins (Ara h 1 and Ara h 6) in Food Matrices. Foods.

[20] Röder M., Wiacek C., Lankamp F., Kreyer J., Weber W., Ueberham E. (2021). Improved Sensitivity of Allergen Detection by Immunoaffinity LC-MS/MS Using Ovalbumin as a Case Study. Foods.

[21] Allred L.K., Nye-Wood M.G., Colgrave M.L. (2020). Analysis of Gluten in Dried Yeast and Yeast-Containing Products. Foods.

[22] Sagu S.T., Zimmermann L., Landgräber E., Homann T., Huschek G., Özpinar H., Schweigert F.J., Rawel H.M. (2020). Comprehensive Characterization and Relative Quantification of α-Amylase/Trypsin Inhibitors from Wheat Cultivars by Targeted HPLC-MS/MS. Foods.

[23] Nugraha R., Ruethers T., Johnston E.B., Rolland J.M., O’hehir R.E., Kamath S.D., Lopata A.L. (2021). Effects of Extraction Buffer on the Solubility and Immunoreactivity of the Pacific Oyster Allergens. Foods.

